# Ageing in Adults With Mild and Moderate Intellectual Disabilities in Brazil: A Biopsychosocial Comparison of Individuals With and Without Down Syndrome

**DOI:** 10.1111/jir.70151

**Published:** 2026-07-28

**Authors:** Leila Regina de Castro, Rita de Cassia Ernandes, Erika Christina Gouveia e Silva, Luciana Mascarenhas Fonseca, José Maria Montiel, Angelica Castilho Alonso

**Affiliations:** ^1^ Program in Aging Sciences Universidade São Judas Tadeu (USJT) São Paulo São Paulo Brazil; ^2^ Jô Clemente Institute São Paulo São Paulo Brazil; ^3^ The Krieger Klein Alzheimer's Research Center, Brain Health Institute Rutgers University New Brunswick New Jersey USA

**Keywords:** ageing, biopsychosocial aspects, Down syndrome, intellectual disability

## Abstract

**Objectives:**

The objective of this study is to investigate functional, cognitive, physical and nutritional outcomes in adults with intellectual disabilities (IDs), comparing individuals with and without Down syndrome from São Paulo, Brazil, and to evaluate the associations of group, degree of disability, age and sex with these outcomes. Additionally, we hypothesized that individuals with Down syndrome would present greater vulnerability to cognitive and physical decline compared to those with ID of other aetiologies.

**Method:**

This cross‐sectional observational study included 52 adults with ID divided into two groups: (1) 24 participants with Down syndrome (ID‐DS) and (2) 28 participants with non‐DS aetiologies (ID‐nonDS). Assessments included sociodemographic and clinical data; functional independence measure (FIM); physical performance (handgrip strength, 30‐s sit‐to‐stand test, gait speed test and MiniBESTest); nutritional status (Mini Nutritional Assessment [MNA]); cognitive function (Cambridge Cognitive Examination for Mental Disorders of the Elderly–Down Syndrome [CAMCOG‐DS]); cognitive decline (Informant Questionnaire on Cognitive Decline in the Elderly [IQCODE]); and functional activities (Pfeffer Questionnaire [QPAF]). Data were obtained through self‐report whenever possible, and proxy report (caregivers) when necessary, based on participants' communication abilities.

**Results:**

Compared with the ID‐nonDS group, the ID‐DS group demonstrated significantly poorer learning (*β* = −2.56; *p* = 0.049) and remote memory performance (*β* = −1.18; *p* = 0.011), a nonsignificant trend towards lower handgrip strength (*β* = −4.68; *p* = 0.051) and more falls (*β* = −0.39; *p* = 0.050). Sex and degree of disability were associated with several outcomes, whereas age showed no significant effect.

**Conclusion:**

Adults in the ID‐DS group exhibited cognitive and physical profiles suggestive of increased vulnerability to age‐related functional impairment compared with adults with other forms of ID. These findings should be interpreted cautiously due to the cross‐sectional design and sample size limitations. Replication in larger samples will be necessary to confirm these observations.

## 1 Introduction

Premature ageing in adults with intellectual disabilities (IDs) is an emerging and challenging phenomenon, still poorly explored in Brazil (Bermudez et al. 2023; Fonseca, Haddad, et al. 2019; Fonseca, Mattar, et al. 2019). It is estimated that 18.6 million Brazilians have some form of disability, and over one billion people worldwide live with significant functional limitations (WHO 2022; World Bank 2020). With population ageing and the rising prevalence of chronic noncommunicable diseases, such as diabetes and cardiovascular disorders, understanding the ageing process in this population has become urgent (WHO 2022).

ID is heterogeneous and may result from genetic, congenital or behavioural factors during gestation or environmental influences, such as birth complications and prematurity (Klein Haneveld et al. 2025; AAIDD 2021; Schalock et al. 2010). Adults with ID with and without Down syndrome exhibit significant limitations in intellectual functioning and adaptive behaviour, affecting social and practical skills essential for daily life (AAIDD 2021). Other factors, including geographic origin, culture and lifestyle, also influence diagnosis and development (Schalock et al. 2010).

In adults with intellectual disability without Down syndrome (ID‐nonDS), ageing often manifests as gradual functional decline, impaired daily activities and communication, as well as behavioural, emotional and health changes (Esbensen et al. 2008). Adults with Down syndrome (ID‐DS), however, are known to present earlier age‐related changes in cognition, physical function and health status compared with adults with ID‐nonDS. These changes include an increased risk of dementia, late‐onset epilepsy and a higher prevalence of endocrine, cardiac, musculoskeletal and sensory comorbidities (Bermudez et al. 2023; Bayen et al. 2018; Fonseca, Mattar, et al. 2019). These differences underscore the need for specialized daily care and individualized interventions tailored to the specific risks and support needs of each group (Evenhuis 2000).

Down syndrome constitutes a distinct biological model of ageing, characterized by well‐established early neurodegenerative processes, thereby justifying its comparison with other aetiologies of ID (Bermudez et al. 2023; Bayen et al. 2018; Fonseca, Mattar, et al. 2019). Nonetheless, individuals with ID‐nonDS represent a clinically and aetiologically heterogeneous group, a factor that must be carefully considered when interpreting comparative findings (Esbensen et al. 2008). This heterogeneity may introduce variability in clinical presentation and outcomes, representing an important limitation when establishing direct comparisons between groups.

Beyond clinical conditions, social inclusion plays a pivotal role in shaping autonomy and overall well‐being among individuals with IDs (Balogh et al. 2010; Santos et al. 2021). Although social and environmental determinants are recognized as influential in the ageing process, the present study primarily addresses clinical and functional dimensions, which should be taken into account when considering the scope and interpretation of the findings. Therefore, broader social determinants are acknowledged but not directly measured in this study, and their potential influence is considered in the interpretation of results.

Despite the growing body of international literature, there remains a significant gap in understanding the biopsychosocial aspects of ageing in adults with IDs in low‐ and middle‐income countries, particularly in Brazil. Furthermore, few studies have simultaneously examined cognitive, physical, functional and nutritional domains in this population using a multidimensional approach.

Drawing on previous literature, we hypothesized that (1) individuals with ID‐DS would exhibit greater cognitive impairment, particularly in memory‐related domains; (2) individuals with ID‐DS would demonstrate poorer physical performance and an increased risk of falls; and (3) these group differences would remain significant after controlling for sex and degree of ID.

Therefore, the present study aimed to investigate the biopsychosocial dimensions of ageing in adults with IDs by comparing individuals with and without Down syndrome. Specifically, we aimed to examine differences in cognitive, physical, functional and nutritional outcomes between groups and to evaluate the associations of group, degree of disability, age and sex with these outcomes.

## 2 Methods

### 2.1 Study Design and Participants

This was a cross‐sectional observational study approved by the Research Ethics Committees (CEP) of the Jô Clemente Institute and São Judas University (CAEE 29093120.5.0000.8647 and CAEE 29093120.5.3001.0089, respectively) and conducted in accordance with the Declaration of Helsinki. In accordance with the Brazilian Inclusion Law (LBI), which recognizes the legal capacity of people with IDs to provide consent, written informed consent was obtained directly from participants, when possible. In cases where participants could not provide consent, it was obtained from their parents or guardians, who signed the Informed Consent Form (ICF), and participant assent was also obtained.

Participants were recruited from a single specialized centre, characterizing a convenience sample. A total of 128 individuals, of both sexes and aged over 35, attending the Jô Clemente Institute, a reference centre for the care of adults with ID in São Paulo, Brazil, were selected and contacted by telephone. Of these, 49 declined to participate due to health restrictions at the time (Covid‐19 pandemic), 20 did not respond and seven withdrew for personal reasons. Fifty‐two adults with ID of both sexes were included, divided into two groups: adults with Down syndrome (ID‐DS) (*n* = 24) and adults with ID‐nonDS (*n* = 28).

Regarding communication skills, no formal ‘a priori’ criteria were established. Instead, the determination of whether a participant would provide self‐reports or require a proxy was based on clinical and functional assessment during the initial contact. Participants who demonstrated difficulty understanding basic instructions or providing consistent responses were assisted by their respective informants to ensure data reliability.

The classification of degree of ID (mild and moderate) was based on ICD codes (F70 and F71), as documented in clinical records at the institution. These records are established through standardized multidisciplinary assessments conducted by the institution's clinical team, following the diagnostic criteria of the International Classification of Diseases (ICD‐10). This documental approach ensures that the classification reflects long‐term clinical monitoring and standardized diagnostic protocols. Although widely used in clinical settings, this classification may not fully capture the functional and cognitive heterogeneity of participants, and this limitation is acknowledged.

The inclusion criteria were diagnosis of ID of genetic origin (Down's syndrome, classified by the International Code of Diseases [ICD] = Q90) and those with ID resulting from other factors (environmental and/or behavioural, without DS) and, for both groups, mild to moderate degree of ID (ICD = F70 and F71).

Exclusion criteria were individuals who were unable to answer the questionnaires or perform the tests; individuals without a diagnosis of ID; and individuals with neurological or sensory conditions that could significantly interfere with motor or cognitive assessments (e.g., advanced dementia, severe neurological disorders or uncorrected sensory impairments).

## 3 Procedures

The surveys were conducted in 2021. Due to Covid‐19 social distancing, the administration of instruments was divided into remote/online application (sociodemographic data, MNA and Pfeffer Questionnaire) and face‐to‐face assessment for physical and cognitive tests, as well as the Functional Independence Measure (FIM), following all current health recommendations.

The assessment protocol was conducted in one to two sessions, depending on participant availability, with each session lasting approximately 60–90 min. The order of physical tests was standardized (handgrip strength, chair stand test, gait speed test and MiniBESTest) to minimize fatigue and ensure consistency across participants.

## 4 Instruments

Sociodemographic data were collected through a questionnaire administered to the informant or caregivers. The variables reported included age, sex, degree of disability, income, housing, main caregiver and associated comorbidities.

Data were obtained through self‐report whenever possible. When participants were unable to provide reliable responses due to cognitive or communication limitations, information was obtained from caregivers. This decision was based on a related to communication ability and was applied consistently across instruments.

### 4.1 Functional Independence Measure (FIM)

The FIM assesses functional independence across two dimensions (physical and cognitive), with a total of 18 items. The physical dimension includes 13 items subdivided into personal care, sphincter control, mobility/transfer and locomotion. The cognitive dimension includes five items subdivided into communication and social cognition. Each item is scored from 1 to 7, yielding a total score ranging from 18 to 126 (Riberto et al. 2004).

### 4.2 Handgrip Strength (HGS)

The HGS is measured using a Jamar dynamometer; participants sat on a chair without armrests, feet flat, hips and knees at 90°, shoulders adducted, elbows at 90°, forearms and wrists neutral. Both hands were tested alternately, with 1‐min rest between trials; the mean of three attempts per hand was recorded in kilogramme‐force (kg/f) (Alonso et al. 2018).

### 4.3 Chair Stand Test (30 s)

Participants were instructed to perform as many sit‐to‐stand repetitions as possible within 30 s, without the use of upper limb support, starting from a standardized seated position (back supported, feet flat on the floor and arms crossed over the chest), in accordance with established protocols (Rikli and Jones 2013).

### 4.4 Gait Speed Test

Participants were instructed to walk at their usual pace over a 4‐m marked walkway, with clearly defined start and end points. The initial position was standardized, and timing began when the participant initiated movement and stopped upon crossing the end line. Three trials were performed with 1‐min rest intervals, and the mean time was calculated for analysis (Rocco and Fernandes 2020).

### 4.5 MiniBESTest

The MiniBESTest consists of a 14‐item instrument subdivided into four subsystems (anticipatory postural adjustments, reactive postural control, sensory orientation and dynamic gait), with a total score of 28 points, where higher scores indicate better balance performance (Marques et al. 2016).

### 4.6 Mini Nutritional Assessment (MNA)

The MNA identifies the risk of malnutrition. It consists of a screening tool divided into four domains: anthropometric assessment, global assessment, dietary assessment and self‐perception. The total score is categorized as follows: ≥ 24 points indicates adequate nutritional status; 17–23.5 points indicates risk of malnutrition; and < 17 points indicates malnutrition (Machado et al. 2015).

### 4.7 Cambridge Cognitive Test for Mental Disorders of the Elderly–Down Syndrome (CAMCOG‐DS)

This is a neuropsychological screening instrument with a maximum score of 107 points, where higher scores indicate better performance. The test includes eight subscales: orientation, language, memory (remote, recent and new learning), attention, praxis, calculation, abstract thinking and perception (Fonseca, Haddad, et al. 2019).

### 4.8 Informant Questionnaire on Cognitive Decline in the Elderly (IQCODE)

A screening tool for cognitive decline was based on informant report over a 10‐year period. Scores range from ≤ 3 (no change) to 5 (significant decline) (Sanchez and Lourenço 2009; Prado Mattar et al. 2022).

### 4.9 Pfeffer Functional Activities Questionnaire (PFAQ)

PFAQ assesses functionality in instrumental activities of daily living via informant report. Scores range from 0 to 30, with lower scores indicating greater independence and scores ≥ 6 suggesting functional dependence (Dutra et al. 2015).

### 4.10 Statistical Analysis

Categorical variables were compared using the chi‐square test or Fisher's exact test, as appropriate. Normality of continuous variables was assessed using the Shapiro–Wilk test. Continuous variables with normal distribution were compared using the Student's *t* test, whereas nonnormally distributed variables were compared using the Mann–Whitney *U* test. Chi‐square statistics and Mann–Whitney *U* values were reported alongside *p* values to improve transparency and reproducibility of the analyses. Bivariate analyses showing statistically significant associations or trends (*p* ≤ 0.10) were entered into linear regression models. Linear regression models were employed to examine associations between group (ID‐DS vs. ID‐nonDS), degree of disability, age and sex with outcomes related to autonomy, independence, physical performance, nutritional status and cognitive function. Given the exploratory nature of the study and the relatively small sample size, findings close to statistical significance were interpreted with caution and not considered confirmatory. A significance level of *p* < 0.05 was adopted for all analyses. As the analyses were considered exploratory, no correction for multiple testing was applied. All statistical analyses were performed using Jamovi Version 2.4.14.0 (The Jamovi Project, Sydney, Australia).

## 5 Results

### 5.1 Characteristics of the Participants

A total of 52 adults with ID participated in the study, including 28 adults without Down syndrome (ID‐nonDS) and 24 adults with Down syndrome (ID‐DS). The ID‐nonDS group presented a higher mean age than the ID‐DS group (47.0 ± 7.7 vs. 43.0 ± 6.5 years, respectively; *p* = 0.050). Regarding the degree of disability, most participants in the ID‐nonDS group were classified as having mild ID (71.4%, *n* = 20), whereas the majority of participants in the ID‐DS group were classified as having moderate ID (79.2%, *n* = 19; *p* < 0.001).

Significant differences were also observed in the source of information used for data collection (*p* = 0.007). Self‐reporting was more frequent among participants in the ID‐nonDS group (50.0%, *n* = 14), whereas most participants in the ID‐DS group required proxy‐reports provided by parents or guardians (87.5%, *n* = 21). Overall, 17 participants (32.7%) completed the assessments independently, whereas 35 (67.3%) required proxy respondents due to communication difficulties and limitations associated with the degree of ID identified during the clinical assessment.

No significant differences were observed between groups for sex, caregiver characteristics, family income, housing conditions, health insurance, medication use, regular medical follow‐up, number of comorbidities or household composition (all *p* > 0.05), indicating that the groups were generally comparable except for differences in disability severity and assessment respondent type (Table 1).

**TABLE 1:** Sociodemographic characteristics of participants with intellectual disabilities (ID) without Down syndrome (ID‐nonDS) and adults with ID and Down syndrome (ID‐DS).

	ID‐nonDS	ID‐DS	*p* value
	Mean (SD)		
Age (years)	47.0 (7.7)	43.0 (6.5)	0.050
Age of caregivers (years)	64.9 (11.2)	61.1 (12.0)	0.249
Number of comorbidities	3.8 (3.3)	2.6 (1.8)	0.128
Household composition (number of residents per household)	2.6 (1.0)	3.1 (1.4)	0.105

	ID‐nonDS group	ID‐DS group	Chi‐square/Fisher
	*n* (%)	*n* (%)	*p* value
Sex, female^a^	10 (35.7)	12 (50.0)	0.400
Degree of disability^a^			
Mild	20 (71.4)	5 (20.8)	< 0.001*
Moderate	8 (28.6)	19 (79.2)	
Regular medical follow‐up, yes^a^	23 (82.1)	23 (95.8)	0.141
Use of medication, yes^a^	18 (72.0)	18 (66.7)	0.455
Health plan, yes^a^	9 (32.1)	16 (66.7)	0.199
Degree of kinship^b^			
Parents	16 (57.1)	12 (50.0)	0.876
Siblings	10 (35.7)	10 (41.7)	
Other family members	2 (7.1)	2 (8.3)	
Planned caregiver in primary informant's absence^b^			
Siblings	19 (67.9)	17 (70.8)	0.111
Other family members	3 (10.7)	6 (25.0)	
Unspecified	6 (21.4)	1 (4.2)	
Monthly family income (minimal Brazilian wages)^b^, ^c^			
1–2	14 (50.0)	9 (37.5)	0.731
3–4	9 (32.1)	8 (33.3)	
5–7	4 (14.3)	5 (20.8)	
≥ 8	1 (3.6)	2 (8.3)	
Housing conditions^b^			
Own	22 (78.6)	18 (75.0)	0.803
Rented	3 (10.7)	2 (8.3)	
Housing provided by government	3 (10.7)	4 (16.7)	
Assessment respondent^b^			
Self‐report	14 (50.0)	3 (12.5)	0.007*
Proxy‐report (parent/guardian)	14 (50.0)	21 (87.5)	

*Note:* For quantitative variables, Student's *t* test.

Abbreviations: %, percentage; ID‐DS, adults with intellectual disabilities with Down syndrome; ID‐nonDS, adults with intellectual disabilities without Down syndrome; *n*, number of participants; SD, standard deviation.

^a^ Chi square test.

^b^ Fisher's test.

^c^ One Brazilian minimum wage (2021) ≈ R$1.621.00 per month, ~300 USD.

* *p* < 0.05 was considered.

### 5.2 Autonomy, Independence, Physical Performance, Nutritional Status and Cognitive Function

Assessment using the Functional Independence Measure (FIM) revealed a significant impairment in the communication domain in the ID‐DS group compared to the ID‐nonDS group (*p* = 0.03). Regarding physical performance, handgrip strength was significantly lower in the ID‐DS group (median: 13.7 vs. 14.2 kg/f; *p* = 0.01), whereas no significant differences were observed in the sit‐to‐stand or gait speed tests (*p* > 0.05). Regarding balance, measured by the MiniBESTest, individuals with ID‐DS demonstrated lower scores in the sensory orientation domain, although this difference did not reach statistical significance (*p* = 0.05). Importantly, both groups presented total MiniBESTest scores below the established cut‐off of 19.5 points, indicating an increased risk of falls across the entire sample.

Nutritional status and anthropometric measures, including BMI and Mini Nutritional Assessment scores, did not differ significantly between groups (*p* > 0.05) (Table 2).

**TABLE 2:** Comparison of autonomy, independence, physical performance, nutritional status and anthropometric measures in adults with ID with and without Down syndrome.

	ID‐nonDS group	ID‐DS group	*p* value
	Median (Q1–Q3)	Median (Q1–Q3)	
Functional independence measure			
Self‐care	41.0 (39.8–41.0)	41.0 (38.8–41.0)	0.27
Sphincter control	14.0 (14.0–14.0)	14.0 (14.0–14.0)	0.71
Mobility transfer	21.0 (21.0–21.0)	21.0 (21.0–21.0)	0.56
Locomotion	13.5 (12.8–14.0)	13.0 (12.8–14.0)	0.76
Communication	14.0 (13.0–14.0)	13.0 (12.0–14.0)	0.03*
Cognition	18.0 (16.0–19.3)	18.0 (14.8–20.0)	0.31
Score total	119.0 (116.0–121.0)	119.0(113.0–121.0)	0.38
Physical performance			
Handgrip strength (kg/f)	17.0 (11.7–19.3)	10.7 (8.4–16.7)	0.01*
Sit and stand 30″	11.5 (10.8–14.0)	10.9 (9.7–13.0)	0.10
Gait speed (s)	5.3 (3.0–7.1)	4.3 (3.0–6.6)	0.90
Mini Best Test			
Anticipatory postural adjustments	4.0 (3.7–4.0)	4.0 (3.7–4.0)	1.00
Reactive postural responses	4.0 (3.0–6.0)	3.5 (0–6.0)	0.21
Sensory orientation	6.0 (6.0–6.0)	6.0 (3.7–6.0)	0.06
Gait stability	8.0 (6.7–9.0)	7.0 (5.7–8.2)	0.24
Total score	22.5 (19.5–25)	20.0 (15–23)	0.08
Mini Nutritional Assessment			
Screening	12.0 (11.0–13.3)	13.0 (12.0–13.0)	0.39
Global assessment	14.0 (13.4–14.6)	14.0 (12.9–15.0)	0.67
Score total	26.5 (24–28)	26.0 (25–27.3)	0.76
Anthropometry			
BMI (kg/m^2^)	28.7 (26.0–32.4)	28.7 (25.1–32.4)	0.73

Abbreviations: BMI, body mass index; ID‐DS group, adults with intellectual disabilities with Down syndrome; ID‐nonDS group, adults with intellectual disabilities without Down syndrome.

Mann–Whitney *U* *p < 0.05.

### 5.3 Cognitive Function and Functional Performance

Cognitive assessment using CAMCOG‐DS demonstrated that adults with ID‐nonDS performed better across all domains compared to those with ID‐DS (*p* ≤ 0.001 across domains). Functional autonomy assessed via the Pfeffer Questionnaire (QPAF) also indicated lower independence in the ID‐DS group (*p* = 0.003). Informant‐reported IQCODE scores remained < 3 in both groups, indicating no clinically significant cognitive decline. However, participants in the ID‐nonDS group showed significantly lower scores (indicating better perceived stability or improvement over time) compared with the ID‐DS group (*p* ≤ 0.001) (Table 3).

**TABLE 3:** Comparison of cognitive function and daily functioning between adults with ID with and without Down syndrome.

	ID‐nonDS group	ID‐DS group	*p* value
	Median (Q1–Q3)	Median (Q1–Q3)	
CAMCOG			
Orientation	9.5 (7.7–12.0)	7.5 (4.0–10.0)	0.001*
Language comprehension	6.0 (5.0–7.5)	5.0 (3.0–6.0)	0.001*
Language expression	15.0 (13.0–16.0)	11.0 (9.0–14.3)	< 0.001*
Language total	21.0 (18.5–23.0)	16.0 (12.0–20.3)	< 0.001*
Memory learning	13.0 (10.8–15.0)	9.5 (5.7–11.0)	< 0.001*
Remote memory	4.0 (2.7–4.0)	2.0 (0–3.0)	0.001*
Recent memory	2.5 (0–4.0)	0 (0–2.2)	< 0.001*
Memory total	19.0 (15.8–21.0)	10.5 (6.7–16.3)	< 0.001*
Attention	9.0 (6.5–9.0)	6.5 (4.7–9.0)	0.001*
Praxis drawing copying	5.0 (3.7–6.0)	4.0 (2.0–5.2)	0.001*
Praxis motor action	8.5 (7.0–9.0)	7.0 (5.0–9.0)	0.003*
Praxia score total	16.0 (10.0–16.0)	14.0 (8.0–14.3)	< 0.001*
Abstract thinking	2.0 (0–3.0)	1.0 (0–3.0)	0.032*
Perception	5.0 (4.0–6.0)	5.0 (3.0–6.2)	0.005*
CAMCOG total	89.0 (72.8–102.0)	68.0 (43.0–90.3)	< 0.001*
IQCODE total	2.0 (1.0–3.0)	2.0 (2.0–3.0)	0.012*
QPAF total	9.0 (3.0–15.8)	14.0 (9.5–19.3)	0.003*

*Note:* CAMCOG score = maximum of 107 points; IQCODE score ≤ 3 no change; 4 considerable change; 5 considerable change; Pfeffer Questionnaire Score (QPAF) = score ranges from 0 to 30; < score greater independence and autonomy.

Abbreviations: CAMCOG, Cognitive Assessment the Cambridge Cognitive Test for Mental Disorders of the Elderly; ID‐DS group, adults with intellectual disabilities with Down syndrome; ID‐nonDS group, adults with intellectual disabilities without Down syndrome; IQCODE, Informant Questionnaire on Cognitive Decline in the Elderly; QPAF, Pfeffer Functional Activities Questionnaire.

Mann–Whitney *U* *p < 0.05.

### 5.4 Associations Between Group, Sex, Degree of IDs and Physical and Cognitive Outcomes

Linear regression analyses revealed that group differences were significantly associated with cognitive outcomes. Individuals with ID‐DS demonstrated poorer performance in learning memory (*β* = −2.56; *p* = 0.049) and remote memory (*β* = −1.18; *p* = 0.011) compared to the ID‐nonDS group. Additionally, borderline associations (‘trends’) were observed for lower handgrip strength (*β* = −4.68; *p* = 0.051) and a higher number of falls (*β* = −0.39; *p* = 0.050) in the ID‐DS group. These findings should be interpreted with caution due to the limited sample size and the proximity of *p* values to the statistical significance threshold.

Among the other predictors, sex was significantly associated with handgrip strength (*β* = 7.20; *p* < 0.001), number of falls (*β* = −0.36; *p* = 0.033) and remote memory (*β* = −0.97; *p* = 0.014), with male participants demonstrating better performance across these outcomes. Degree of ID was positively associated with learning memory (*β* = 3.07; *p* = 0.016) and remote memory (*β* = 1.06; *p* = 0.017). Age was not significantly associated with any of the analysed outcomes (*p* > 0.05), a finding that should be interpreted in light of the relatively restricted age range and the cross‐sectional design of the study (Table 4).

**TABLE 4:** Linear regression analysis of group, sex, age and degree of intellectual disabilities on physical and cognitive outcomes in adults with intellectual disabilities with and without Down syndrome.

Handgrip strength	Estimates	Standard error	*t*	*p*	Adjusted *R* ^2^
Intercept	10.4068	7.527	1.383	0.173	0.304
Group: ID‐DS–ID‐nonDS	−4.6765	2.338	−2.000	0.051	
Degree (of disability)	−0.5126	2.272	−0.226	0.822	
Age (years)	−0.0946	0.137	−0.692	0.492	
Sex (M‐F)	7.2045	1.993	3.615	< 0.001	
Falls					
Intercept	1.40504	0.6193	2.269	0.028	0.178
Group: ID‐DS–ID‐nonDS	−0.38769	0.1924	−2.015	0.050	
Degree (of disability)	−0.32106	0.1870	−1.717	0.093	
Age (years)	−0.00179	0.0112	−0.159	0.874	
Sex (M‐F)	−0.36002	0.1640	−2.195	0.033	
Learning memory					
Intercept	14.5970	4.0761	3.5811	< 0.001	0.314
Group: ID‐DS–ID‐nonDS	−2.5593	1.2664	−2.0210	0.049	
Degree (of disability)	3.0740	1.2305	2.4981	0.016	
Age (years)	−0.0908	0.0740	−1.2268	0.226	
Sex (M‐F)	−0.0124	1.0794	−0.0115	0.991	
Remote memory					
Intercept	3.90218	1.4266	2.735	0.009	0.394
Group: ID‐DS–ID‐nonDS	−1.18251	0.4459	−2.652	0.011	
Degree (of disability)	1.06006	0.4293	2.469	0.017	
Age (years)	0.00315	0.0262	0.120	0.905	
Sex (M‐F)	−0.96969	0.3802	−2.551	0.014	

*Note:* Degree of disability was coded as 0 = moderate intellectual disability and 1 = mild intellectual disability.

Abbreviations: β, regression coefficient; ID‐DS, adults with intellectual disabilities with Down syndrome; ID‐nonDS, adults with intellectual disabilities without Down syndrome; M‐F, male versus female; *R* ^2^, adjusted–adjusted coefficient of determination.

## 6 Discussion

In this sample of 52 adults with ID with and without DS from São Paulo, Brazil, we found that adults with ID‐DS exhibited poorer cognitive performance, particularly in learning and remote memory, compared with adults with ID‐nonDS. These findings are consistent with our a priori hypotheses and support the notion of a differential ageing profile associated with Down syndrome. Trends towards lower handgrip strength and a higher number of falls were also observed in the ID‐DS group, although these did not reach statistical significance in the adjusted models. Given the limited sample size and the proximity of *p* values to the significance threshold, these borderline findings should be interpreted with caution. Unadjusted comparisons further indicated differences in communication, handgrip strength and overall cognitive domains. Among the other predictors, sex was associated with better handgrip strength, fewer falls and higher remote memory scores in men. Additionally, participants with mild ID demonstrated better performance in learning and remote memory than those with moderate ID, suggesting greater baseline cognitive reserve. Contrary to expectations based on previous literature, age was not significantly associated with any of the outcomes, which may reflect the relatively restricted age range and the cross‐sectional design of the study.

Collectively, these findings reinforce the presence of specific cognitive vulnerabilities and emerging physical limitations in ageing adults with Down syndrome, while also underscoring the influence of demographic and disability‐related factors on functional and cognitive performance in this population. Importantly, the observed differences between groups should be interpreted in light of the heterogeneity of the ID‐nonDS group, which may encompass individuals with diverse clinical and aetiological profiles, potentially contributing to increased variability in outcomes and attenuating between‐group contrasts. Although cognitive findings were consistent with the study hypotheses, physical outcomes showed only borderline associations, suggesting that these domains may be more sensitive to sample size limitations and measurement variability.

The assessment of physical performance indicated that adults with ID‐DS had lower handgrip strength compared with adults with ID‐nonDS. Regression analysis showed that female sex was also associated with lower strength values, whereas ID‐DS demonstrated a trend towards reduced strength. These findings are consistent with Mehra et al. (2025), who reported that adults with ID‐DS exhibit additional reductions in strength, lean mass and muscle quality, and with Hilgenkamp et al. (2012), who noted that even adults with ID‐nonDS already have lower strength than the general population. Reduced handgrip strength is a well‐established marker of ageing, associated with frailty, functional decline and increased risk of morbidity and mortality (Alonso et al. 2018). Therefore, even modest reductions observed in this study may have clinically meaningful implications, particularly in populations already vulnerable to functional decline.

Assessment of postural balance revealed that adults in both groups scored below the MiniBESTest cut‐off of 19.5, classifying them as at risk for falls (Pope et al. 2020; Doherty et al. 2023). Regression analysis indicated a trend towards a higher number of falls in the ID‐DS group, whereas female sex was significantly associated with falls, highlighting women with ID as particularly vulnerable. Adults with ID‐DS tended to show poorer performance in the sensory orientation subdomain of the MiniBESTest compared with adults with ID‐nonDS, in line with previous studies, although this difference did not reach statistical significance. This pattern should be interpreted cautiously, as the absence of statistical significance may reflect limited statistical power rather than the absence of a true effect.

This trend likely reflects phenotype‐related factors such as hypotonia, ligament laxity, orthopaedic issues, obesity, sensory impairments (vision and hearing), seizures and slower neurological processing (Benraad et al. 2022). The combination of reduced handgrip strength and impaired postural balance contributes to immobility and instability, which are key components of the so‐called ‘I's’ of geriatric syndromes—a set of multifactorial conditions including reduced mobility, balance impairment and cognitive decline associated with functional dependence.

In adults with ID, particularly those with ID‐DS, these vulnerabilities may be further exacerbated by contextual and clinical factors, including sedentary behaviour, obesity, comorbidities (mean 2.6 ± 1.8) and medication use (66.7%), potentially accelerating physical decline. Moreover, limited awareness of fall risks and lack of use of assistive devices may further increase vulnerability to falls and functional deterioration.

Regression analyses revealed significant deficits in learning and remote memory in adults with ID‐DS compared with those with ID‐nonDS, even after controlling for age and degree of disability. This pattern suggests a profile consistent with specific cognitive vulnerability rather than definitive evidence of accelerated ageing, given the cross‐sectional nature of the study. Although accelerated ageing implies a premature but otherwise typical decline, our findings point towards a ‘double hit’ phenomenon: an atypical neurodevelopmental baseline coupled with an early, domain‐specific susceptibility of the temporal and frontal lobes. This vulnerability may render individuals with Down syndrome more sensitive to the initial stages of amyloid deposition, leading to the observed deficits in learning and remote memory even before the onset of global functional decline. These findings may be interpreted in the context of prior evidence indicating that cognitive decline in individuals with ID‐DS follows a domain‐specific trajectory, in which deficits in working memory, attention and executive functions often precede overt episodic memory loss (Lautarescu et al. 2017).

Regarding the linear regression findings, the positive association between the degree of ID and memory outcomes (learning and remote memory) warrants careful interpretation. Participants classified with a mild degree of ID demonstrated significantly better performance in these cognitive domains compared to those with a moderate degree. This suggests that a higher baseline level of cognitive and functional functioning may act as a protective factor, potentially reflecting greater cognitive reserve within the spectrum of ID. According to the cognitive reserve framework, individuals with greater baseline cognitive resources may be better able to cope with age‐related or neurodegenerative changes, maintaining cognitive performance despite underlying neuropathological processes (Stern et al. 2020). Therefore, the superior memory performance observed among participants with mild ID may reflect a greater capacity to compensate for cognitive decline, whereas individuals with more severe impairments may be more vulnerable to the functional consequences of age‐related cognitive changes.

Previous studies have suggested that the earliest manifestations of dementia in adults with Down syndrome may be subtle and often involve behavioural, executive and functional changes before the emergence of more pronounced memory deficits (Lautarescu et al. 2017; Prado Mattar et al. 2022). In our sample, poorer performance in remote memory was more pronounced among women with DS. Although sex‐related differences in cognitive outcomes have been reported in adults with Down syndrome, findings remain inconsistent across studies and cognitive domains (Lulita et al. 2023). These inconsistencies highlight the need for further investigation of sex‐related factors and their potential influence on cognitive ageing trajectories in this population.

From a functional and clinical perspective, the cognitive alterations observed in this study, particularly in learning and remote memory, may have important implications for autonomy and adaptive functioning. Although communication differences did not remain significant in the adjusted regression models, adults with ID‐DS exhibited poorer cognitive performance than adults with ID‐nonDS. Previous studies have shown that cognitive and communication abilities are closely related to adaptive functioning, independent living skills and participation in daily activities among adults with IDs and Down syndrome (Esbensen et al. 2008; Matthews et al. 2018; Santos et al. 2021). Therefore, the cognitive vulnerabilities identified in the present study may be associated with greater support needs and reduced functional independence, highlighting the importance of early assessment and targeted interventions aimed at preserving autonomy and quality of life in this population.

### 6.1 Limitations

Several limitations should be considered when interpreting the present findings. First, the cross‐sectional design precludes causal inferences regarding the relationships observed between cognitive, functional and psychosocial variables. Therefore, the results should be interpreted as clinically meaningful associations rather than evidence of cause‐and‐effect relationships.

Second, participants were recruited through convenience sampling from a single specialized centre, which may limit the generalizability of the findings to broader populations of adults with ID. This limitation is particularly relevant given the heterogeneity of the ID without Down syndrome (ID‐nonDS) group, which comprised individuals with diverse aetiological backgrounds and may have contributed to greater variability in the outcomes.

Third, the relatively small sample size may have reduced statistical power, potentially contributing to the absence of significant associations in some analyses and to the borderline *p* values observed for certain outcomes. Additionally, no correction for multiple comparisons was applied because of the exploratory nature of the study, increasing the possibility of type I error.

Fourth, although the classification of ID was based on ICD‐10 diagnoses documented in clinical records, this approach may not fully capture the functional, adaptive and cognitive heterogeneity of participants. Moreover, the groups differed significantly in the severity of ID, which may represent a potential source of residual confounding despite adjustment in the regression analyses.

Finally, not all assessment instruments were originally developed or validated for adults with ID, with or without Down syndrome. Although standardized administration procedures and necessary adaptations were implemented to maximize accessibility and comprehension, measurement bias cannot be completely excluded. This limitation highlights the need for future studies to develop and validate assessment tools specifically designed for adults with ID, incorporating accessible language and appropriate psychometric properties.

Longitudinal and multicentre investigations with larger and more representative samples are warranted to confirm these findings and further elucidate the trajectories of ageing, cognition and functional decline in adults with IDs.

### 6.2 Implications and Future Directions

In summary, the present study underscores the importance of an integrated biopsychosocial framework that accounts for the specific clinical and functional profiles of adults with ID‐DS and ID‐nonDS. However, the interpretation of these findings should be situated within the exploratory nature of the study. These results carry relevant clinical and research implications, emphasizing the need for population‐specific assessment approaches and the development of targeted, multidisciplinary intervention strategies. In particular, the adaptation and validation of assessment instruments, alongside the implementation of multifaceted interventions, may play a critical role in mitigating functional decline and optimizing long‐term health outcomes in this population.

Given the cross‐sectional design, these findings should not be interpreted as evidence of accelerated ageing trajectories but rather as indicative of differential vulnerability patterns between groups. Accordingly, future longitudinal studies with larger and more representative samples are warranted to further elucidate ageing trajectories and confirm the patterns observed in this study.

## 7 Conclusion

The findings of this study indicate that adults with ID‐DS exhibit more pronounced impairments in specific cognitive domains, including learning and remote memory, as well as reduced handgrip strength and lower functional communication. Although both groups demonstrated challenges in multiple domains, individuals with ID‐DS presented a profile suggestive of increased vulnerability to functional decline.

## Funding

The authors have nothing to report.

## Conflicts of Interest

The authors declare no conflicts of interest.

## Data Availability

The data that support the findings of this study are openly available in Repositório Universitário da Anima (RUNA) (at https://repositorio.animaeducacao.com.br/handle/ANIMA/26011).
